# Temporal Reinforcement of Naloxone Training Results in Significant Retention of Anti-bias and Overdose-Response Knowledge

**DOI:** 10.7759/cureus.45415

**Published:** 2023-09-17

**Authors:** Pandurang Bharne, Erin Kelly, Sujay Rajkumar, Austin Iglesias, Sravya Ponnapalli, Benjamin Haslund-Gourley, Annette Gadegbeku

**Affiliations:** 1 College of Medicine, Drexel University, Philadelphia, USA

**Keywords:** medical education & training, stigma scale, opioid use disorders, narcan, opioid use

## Abstract

Background: The opioid epidemic is an increasingly severe problem affecting public health and leading to significant economic burdens on healthcare systems. Overdose reversal training and de-stigmatization efforts are common strategies used to combat this epidemic. Nevertheless, healthcare professionals report a lack of confidence in administering naloxone and high stigmatization levels toward people with opioid use disorder (OUD). While one-time educational training reduces stigma and improves naloxone administration confidence, we previously demonstrated that knowledge retention at a three-month follow-up is reduced among a cohort of medical students. This study aimed to improve the effectiveness of opioid overdose awareness and reversal training (OOART) with a three-month follow-up abbreviated OOART (aOOART) booster video.

Methods: Voluntary OOART was offered to first-year medical students (M1) at the Drexel University College of Medicine in 2022. At this training, 82 students completed a pre-training survey to establish a baseline knowledge and attitude toward people with OUD and their familiarity with the steps to reverse an opioid overdose. Following the hour-long training, 64 of 82 (79%) participants completed the post-training survey to measure the immediate retention of training information. After 2.5 months, students were randomly selected to receive a 6.5-minute aOOART booster video or serve as an unboosted control. Students in the booster and non-booster cohorts then completed a three-month follow-up survey.

Results: Students who received the aOOART booster had significantly increased opioid reversal knowledge scores compared to non-boosted control students at the three-month follow-up. The aOOART booster resulted in a retention of the lowered stigma, and participants expressed a higher willingness to respond to an opioid overdose compared to participants who did not receive the booster video at the three-month follow-up.

Conclusions: This study shows that an aOOART booster method improves knowledge retention following initial OOART. Further, the aOOART booster video served to maintain participants’ reduced stigma toward people with OUD and maintained participants’ willingness to respond to an opioid overdose. These results support the incorporation of an abbreviated, periodic OOART reinforcement video into opioid overdose response training nationwide. We believe this “booster video” approach is a novel and effective method to improve healthcare professionals’ and medical student preparedness to render appropriate care to people with OUD.

## Introduction

Opioid misuse and related overdose deaths are a major public health concern in the United States. The opioid crisis is a public health emergency, with over 564,000 fatalities due to opioid overdose between 1999 and 2020 [1,2]. Despite public concern, the number of overdose deaths involving opioids continues to escalate, with nearly 80,000 provisional deaths reported in the 12-month ending period for November 2022 [3]. The prevalence of synthetic opioids, primarily fentanyl and related piperidine-based analogs, drives this escalation in opioid-related deaths [4]. For example, there has been a 1000% increase from 2013 to 2019 in synthetic opioid overdose-related deaths [5]. This is because synthetic opioids are added to heroin to increase potency while reducing the cost [5]. One of the cities in the United States hit the hardest by the opioid epidemic is Philadelphia, PA [6]. In 2021, Philadelphia reported the highest number of unintentional overdoses at 1,276 fatalities, with 82% of those deaths due to opioids [7]. Furthermore, an active compound known as xylazine, a non-opioid veterinary tranquilizer not approved for human use, has become much more prevalent in the illicit drug supply in Philadelphia. Between 2010 and 2015, less than 2% of fatal heroin and/or fentanyl overdoses contained xylazine; by 2019, this rose to 31% [8]. Taken together, it is important to address the opioid overdose crisis from new angles, implement harm reduction, and provide more accessible care [9].

One of the best tools for harm reduction is the medication naloxone. Naloxone (also known as Narcan) is a pure mu-opiate receptor competitive antagonist that can reverse opioid overdoses when given intranasally, intramuscularly, or intravenously [10]. Naloxone is recognized as an effective measure to prevent overdose fatalities due to ease of use, particularly when delivered intranasally. Communities where recreational use is present have demonstrated to benefit from peer-administered naloxone because peers often have access to high-risk or hard-to-reach drug scenes [11,12]. Naloxone standing orders permit any person to obtain naloxone without the prescription of a physician via pharmacy-based naloxone (PBN) distribution [13,14] and have substantially increased access to naloxone [15]. While access to and distribution of naloxone are initial steps in addressing the opioid crisis, this is not sufficient on its own to combat the opioid epidemic [16]. It is imperative to further increase the awareness of naloxone availability and broaden education in naloxone administration to reduce the stigma around naloxone use as a harm reduction method. If patients who are rescued from an opioid overdose via naloxone are treated with high levels of stigma by their healthcare team, then these same patients are more likely to continue to struggle with opioid use disorder (OUD) rather than receive the emotional and systemic support required to overcome OUD [17]. Thus, education of medical professionals can improve their stance on and readiness to provide harm-reduction-centered care. Improvement in attitudes toward overdose reversal and stigma reduction was first demonstrated in the training of internal medicine physicians using opioid overdose awareness and reversal training (OOART) [18]. 

Similar studies have since been reproduced in several pre-professional school programs, including medical students [19-23]. In these studies, OOART has corrected misconceptions, addressed gaps in knowledge, reduced the stigma around opioid overdose reversal using naloxone, and demonstrated a positive change in the confidence and attitudes of the participants. Furthermore, these studies have been reproduced longitudinally to examine retention of naloxone reversal knowledge and positive attitudes toward harm reduction [24,25]. It is imperative that once a person is trained in OOART, they can accurately recall how to administer naloxone and maintain a positive attitude regarding harm reduction. Participants in these studies generally reported positive knowledge retention, but a recent study has found that after a three-month follow-up, participants' confidence in using this knowledge had regressed to near baseline in comparison to confidence immediately after OOART [26]. One method to prevent knowledge regression in medical education is the use of repeated practice for knowledge maintenance [27].

Drexel University College of Medicine's (DUCOM's) students-led Naloxone Outreach Project (NOP) provides comprehensive OOART and take-home naloxone to future healthcare provider students and other members of the Philadelphia community. NOP delivered several in-person training sessions in the fall and winter of 2022 to first-year medical students. All students were given pre-, post-, and three-month-post-training surveys. The students given a three-month-post-training survey were split into two groups, in which one group was also administered an abbreviated booster video to promote knowledge retention while the other group received no follow-up training. The booster video consisted of a short 6.5-minute video file recorded by the same presenters as three months prior. The focus of this study is to expand upon previous findings and provide a solution to the knowledge and attitude regression shown in previous studies after three months. The objectives of this paper are to (1) determine the efficacy of our OOART and (2) compare knowledge and attitude metrics before, immediately after, and three months after receiving OOART with and without an abbreviated booster video.

## Materials and methods

Development of OOART

A team of medical students at DUCOM, along with support from faculty advisors and members of the Philadelphia Department of Public Health, developed the OOART as outlined in Goss et al. [28]. The group included individuals with personal experience dealing with OUD. This training provides practical knowledge on naloxone administration, as well as unique insights into the sociohistorical development of the opioid epidemic and the biopsychosocial factors relevant to individuals with OUD. The OOART consists of a PowerPoint presentation divided into seven sections, covering (1) Opioid Basics, (2) Introduction to the Opioid Epidemic, (3) A Brief History and the Aftermath, (4) The Experience of OUD, (5) Race/Ethnicity Disparities in the Opioid Epidemic, (6) OUD Treatment and Harm Reduction as a Tool, and (7) Overdose Reversal, Naloxone Administration, and Post-Reversal Care. Section 7 of the presentation includes four videos produced by NOP simulating an overdose scenario, with one student portraying the individual who overdosed and the other acting as the "Good Samaritan" administering the overdose reversal. 

The OOART booster video was also created by the NOP team at DUCOM with support from faculty advisors and members of the Philadelphia Department of Public Health. The booster video is a 6.5-minute refresher reviewing the signs of an overdose, appropriate overdose reversal steps, and post-opioid reversal care, which are all discussed in section 7 of the OOART described above. The booster video (Video 1) is linked here.

**Video 1:** Booster NOP 22-23 Video

OOART Survey

Surveys were administered to DUCOM students before training (pre-training), directly after training (post-training), and after three months (three-month-post-training). The pre-training survey includes demographic information including age, gender, current education level, and employment status. It also asks student participants about prior naloxone training, any experience witnessing an overdose, previous naloxone administration, and whether they currently carry naloxone. The same questions used in the pre-training survey were utilized in the post-training, and three-month-post-training surveys to gather data on attitudes and knowledge retention.

To assess attitudes toward overdoses and OUD, the survey includes 12 questions. These questions are further divided into three subsections: (a) “Attitudes Towards Naloxone and Overdose Reversal,” (b) “Attitudes Towards Individuals with OUD”, and (c) “Self-Confidence in Using Naloxone and Handling an Overdose.” Our modified survey includes six questions pertaining to “Attitudes Towards Naloxone and Overdose Reversal” or “Self-Confidence in Using Naloxone and Handling an Overdose.” Six additional questions were created to address “attitudes towards individuals with OUD.” The exact wording and categorization of the questions are presented in Table 1. All attitude questions are scored on a 5-point Likert Scale (Completely Disagree = 1; Disagree = 2; Unsure = 3; Agree = 4; Completely Agree = 5). To assess knowledge, the survey includes three multiple-choice fact-based questions adapted and shortened from the Opioid Overdose Knowledge Scale [24]. The competency questions are scored as “1” for correct or “0” for incorrect. This allows for comparison between individual questions and between overall percent correct.

**Table 1:** OOART Attitude Question Key

Question Number	Question
Q1	If someone overdoses, I want to be able to help them.
Q2	Everyone should learn how to use and carry naloxone.
Q3	I will do whatever is necessary to save someone's life in an overdose situation.
Q4	It is understandable why those who use drugs and experience withdrawal symptoms may use drugs daily.
Q5	We need to provide ways to keep people alive and minimize the harms associated with drug use (ex: needle exchange programs, naloxone distribution) to effectively deal with the opioid epidemic.
Q6	People often start using opioids and find it hard to quit due to a lack of willpower and discipline.
Q7	It is understandable that many people are not ready, willing, or able to get treatment for substance use disorders.
Q8	My attitudes toward people who use drugs, and how I think and talk about them, has nothing to do with their ability to seek or receive help.
Q9	I would be afraid of doing something wrong in an overdose situation.
Q10	If I saw an overdose, I would panic and not be able to help.
Q11	I would be able to deal effectively with an overdose.
Q12	Racism and stigma create disparities in US federal and societal responses to substance use disorder.
Q-all Positive	Summation of attitude questions that were anticipated to have scores of 4 or 5 (agree or strongly agree) denoted in bold above
Q-all Negative	Summation of attitude questions that were anticipated to have scores of 1 or 3 (disagree or strongly disagree) denoted as regular text above

Delivery of OOART and booster video

Trainings were conducted in person in Philadelphia in October and November 2022. Participation in the training was voluntary, and participants included first-year medical students and students in other graduate programs of Drexel University. Pre-surveys were conducted through a QR code before the beginning of the training session. Participants were instructed to complete the survey prior to the presentation. At the end of the session, participants completed the post-survey using a link provided in a follow-up email. Two and a half months after the online OOART, half of the participants were emailed a 6.5-minute booster video, while the other half of the participants were not emailed the booster video and served as the control group. Two weeks after the booster video was received by the students, a three-month-post-training survey was distributed to all participants via email. As with many other opioid overdose prevention programs, naloxone was distributed to all participants who completed the pre- and post-training surveys.

Data analysis

Analysis for statistical differences between surveys was conducted using a nonparametric one-way analysis of variance (ANOVA), with post hoc Tukey's multiple-comparisons test to examine the three time points for statistically significant differences. The calculated p-values of < 0.05 were considered statistically significant. Descriptive statistics were also used to display the pre-, post-, three-month-post-non-boosted, three-month-post-boosted, and mean and standard deviation for each question. Figures are generated using GraphPad Prism Software Version 8 (GraphPad Software, San Diego, CA).

Recruitment and inclusion/exclusion criteria

Medical and graduate students were recruited predominantly through emails and by word of mouth. All participation was voluntary. There were no specific inclusion criteria. All demographic data were included in the results, regardless of whether the remainder of the survey was complete or incomplete. This research was conducted under Drexel’s Health Outreach Program (HOP) IRB Protocol #: 1904007126. Furthermore, this research assesses student performance on surveys in a de-identified manner and thus does not constitute human subjects research according to United States Department of Health and Human Services Regulation 45 CFR 46.

OOART survey

Twelve attitude questions are included in the pre-, post-, and three-month-post-OOART surveys (Table 1). We classify these questions by the expected positive or negative trend in responses following OOART. For example, three attitude questions (Q6, Q9, and Q10) are written to obtain a lower score on the 1-5 Likert scale and assess the fear or latent stigma an OOART participant may harbor toward people with OUD. Another eight attitude questions (Q1, Q2, Q3, Q4, Q5, Q7, Q11, and Q12 denoted with a bolded text in Table 1) are written to obtain a higher score on the 1-5 Likert scale and suggest that an OOART participant holds little to no stigma about people with OUD. In our study, we assume that a higher score in the first group of eight attitude questions suggests the participant is ready to assist in an opioid overdose reversal.

The OOART survey also determines the participants' knowledge of rescue breaths, naloxone pharmacokinetics, and the steps of a successful opioid overdose reversal using seven questions (Table 2). All scores are summed to create a composite of the correct answers, assessing the overall knowledge of each OOART participant before, after, and three months after OOART. 

**Table 2:** OOART Knowledge Question Key OOART, opioid overdose awareness and reversal training.

	Question	Answer
K1	Which of the following is the correct pattern for giving rescue breaths?	2 breaths every 5 seconds
K2	How long does intranasal naloxone last in the average person's system?	30-90 minutes
K3	What is the correct sequence of steps for opioid overdose reversal with naloxone?	[First] Stimulate
K4	What is the correct sequence of steps for opioid overdose reversal with naloxone?	[Second] Call 911
K5	What is the correct sequence of steps for opioid overdose reversal with naloxone?	[Third] Administer Narcan (naloxone)
K6	What is the correct sequence of steps for opioid overdose reversal with naloxone?	[Fourth] Rescue breaths
K7	What is the correct sequence of steps for opioid overdose reversal with naloxone?	[Fifth] Evaluate
K- all	Total scores summed across the seven questions above	

## Results

**Figure 1:**
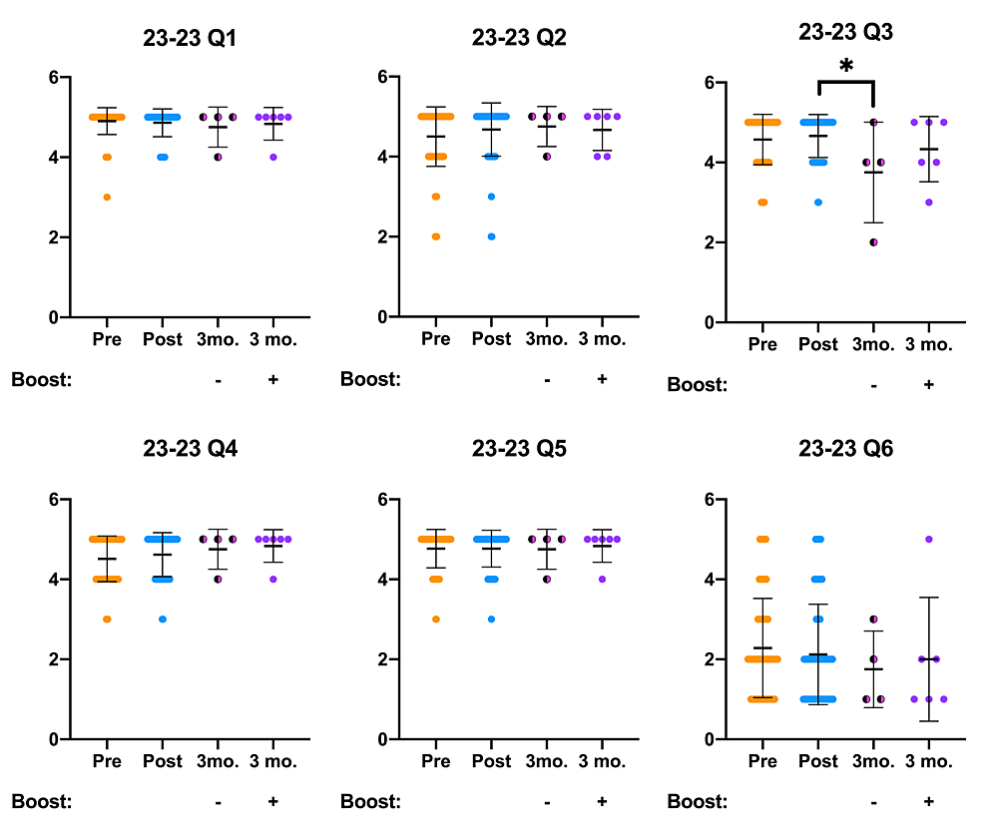
OOART attitude questions (Q1-Q6) are presented from pre-OOART (orange dots), post-OOART (blue dots), three-month-post-OOART without booster training (half-black, half-pink dots), and three-month-post-OOART with booster training (purple dots) Pre-OOART (n = 82), post-OOART (n = 65), three-month-post-OOART (n = 4), and three-month-post-OOART with booster training (n = 6), mean ± standard deviation. A one-way ANOVA with Tukey's multiple-comparisons test was used to determine statistical significance: *p < 0.05. OOART, opioid overdose awareness and reversal training; ANOVA, analysis of variance.

**Figure 2:**
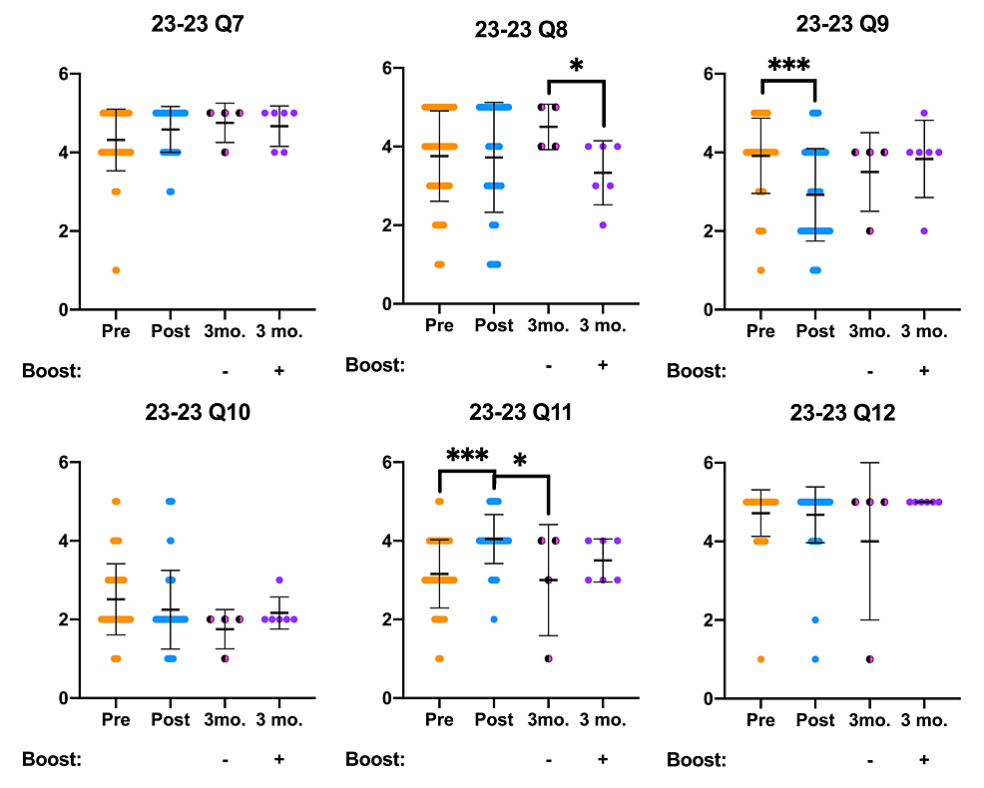
OOART attitude questions (Q7-Q12) are presented from pre-OOART (orange dots), post-OOART (blue dots), three-month-post-OOART without booster training (half-black, half-pink dots), and three-month-post-OOART with booster training (purple dots) Pre-OOART (n = 82), post-OOART (n = 65), three-month-post-OOART (n = 4), and three-month-post-OOART with booster training (n = 6), mean ± standard deviation. A one-way ANOVA with Tukey's multiple-comparisons test was used to determine statistical significance: *p < 0.05, **p < 0.01, ***p < 0.001. OOART, opioid overdose awareness and reversal training; ANOVA, analysis of variance.

OOART participant attitude survey results are presented below and analyzed for statistically significant differences (Figures 1, 2). In addition to the canonical pre- and post-OOART responses, we present a three-month follow-up survey data stratified by a “booster” intervention. Before accessing the three-month follow-up OOART survey, half of the participants received either a 6.5-minute booster video created by the Naloxone Outreach Project at DUCOM or an email inviting them to participate in the follow-up OOART survey. Participants who received the booster video completed the three-month follow-up survey two weeks later. Attitude responses from the non-boosted three-month follow-up cohort in Q3, Q8, and Q11 exhibited significantly different Likert scores in comparison to the post-OOART survey responses. On the other hand, three-month follow-up OOART who received the booster intervention responded with Likert scores similar to the post-OOART time point. 

**Figure 3:**
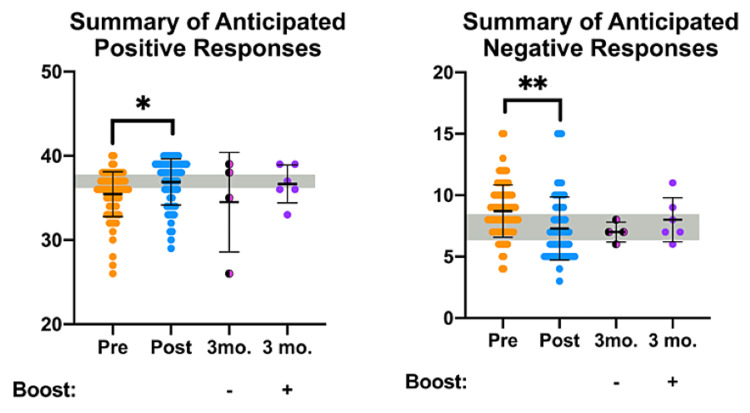
Attitude OOART responses categorized by "positive" and "negative" views of people with OUD or people who experience an opioid overdose are presented from pre-OOART (orange dots), post-OOART (blue dots), three-month-post-OOART without booster training (half-black, half-pink dots), and three-month-post-OOART with booster training (purple dots) Pre-OOART (n = 82), post-OOART (n = 65), three-month-post-OOART (n = 4), and three-month-post-OOART with booster training (n = 6), mean ± standard deviation. A one-way ANOVA with Tukey's multiple-comparisons test was used to determine statistical significance: *p < 0.05 and **p < 0.01. OOART, opioid overdose awareness and reversal training; ANOVA, analysis of variance.

To assess overall trends of OOART participant attitudes, we combined the questions grouped by their expected positive or negative trends. We refer to these groups as Q-All Positive and Q-All Negative in Table 1 and present the resulting data in Figure 3. The summary of the anticipated positive pre-OOART survey responses significantly improved at the post-OOART time point (gray bar denoting the target response) as previously reported [26]. At the three-month follow-up, participants who received the booster video maintained a high average of positive responses with a small standard deviation (purple dots). In comparison, the non-boosted participants exhibited a large standard deviation and a lower average of anticipated positive responses (pink/black dots). Encouragingly, the summary of anticipated negative responses by participants was reduced following OOART, and the average responses in booster and non-boosted three-month follow-up surveys remained low. Taken together, the summary of attitude responses reflects that the booster video promotes the maintenance of positive attitudes toward people with OUD months after the OOART training.

**Figure 4:**
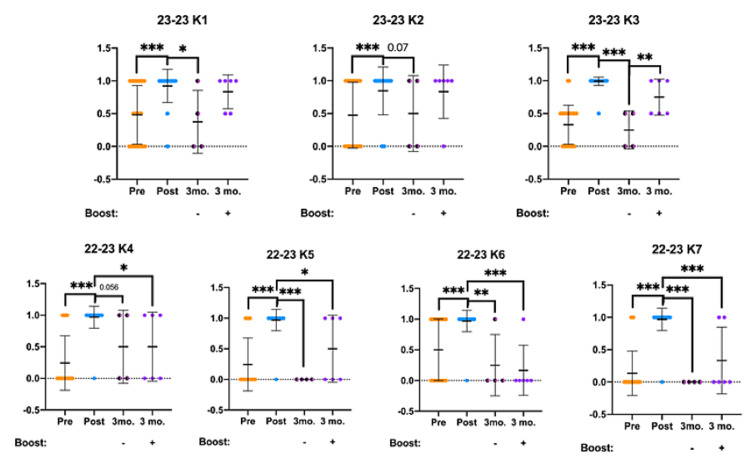
OOART knowledge questions are presented from pre-OOART (orange dots), post-OOART (blue dots), three-month post-OOART without booster training (half-black, half-pink dots), and three-month post-OOART with booster training (purple dots) Pre-OOART (n = 82), post-OOART (n = 65), three-month-post-OOART (n = 4), and three-month-post-OOART with booster training (n = 6), mean ± standard deviation. Please see Table 2 for a full list of question prompts for questions K1-K7. A one-way ANOVA with Tukey's multiple-comparisons test was used to determine statistical significance: *p < 0.05, **p < 0.01, ***p < 0.001. OOART, opioid overdose awareness and reversal training; ANOVA, analysis of variance.

OOART participant knowledge survey results are presented below and analyzed for statistically significant differences (Figure 4). Answers to knowledge questions were broken down into 0 = incorrect, 0.5 half-credit, and 1 = correct scores for this analysis. Significant increases in knowledge scores were observed following the OOART training as previously reported by analyzing the pre- and post-OOART survey results. Fascinatingly, while the boosted three-month follow-up responses maintained a high degree of correct answers compared to the post-OOART scores, the non-boosted three-month follow-up scored significantly lower in questions K1, K3, and K5. However, the boosted three-month follow-up survey responses were also lower, but to a lesser degree in questions K3, K4, K5, K6, and K7. 

**Figure 5:**
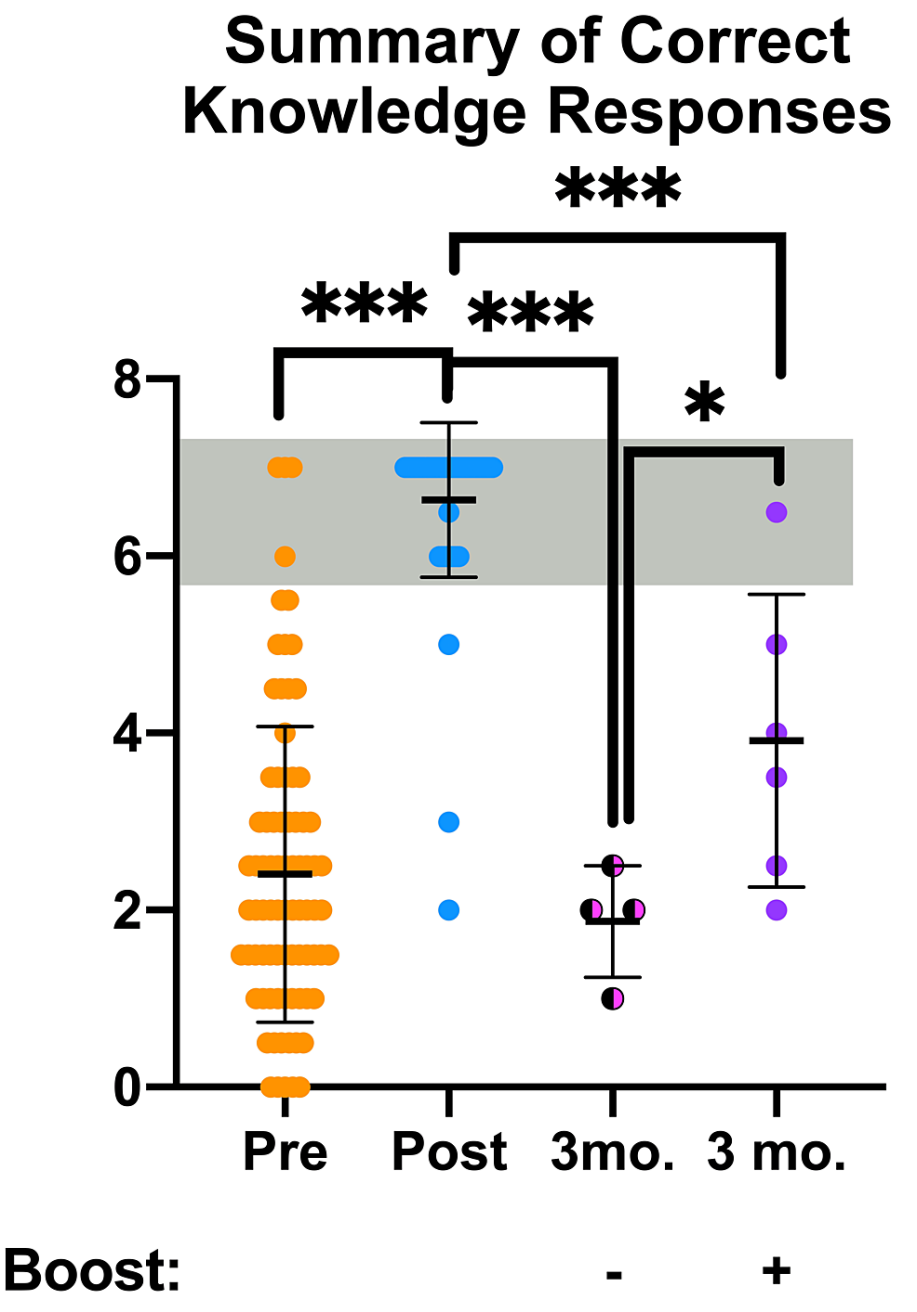
Knowledge OOART responses categorized by the number of correct answers to questions reviewing the steps to reverse an opioid overdose are presented from pre-OOART (orange dots, n = 82), post-OOART (blue dots, n = 65), three-month post-OOART without booster training (half-black, half-pink dots, n = 4), and three-month post-OOART with booster training (purple dots, n = 6), mean ± standard deviation. A one-way ANOVA with Tukey's multiple-comparisons test was used to determine statistical significance: *p < 0.05, **p < 0.01, ***p < 0.001. OOART, opioid overdose awareness and reversal training; ANOVA, analysis of variance.

Last, OOART knowledge responses were combined into an aggregate score to determine the overall participant knowledge and competence to respond to an opioid overdose (Figure 5). As previously reported, the pre- vs. post-OOART scores significantly improved to reach the target range denoted with the gray bar. Examining the scores from the non-boosted three-month follow-up OOART survey revealed a significant loss of knowledge - similar to that of a participant who had not received OOART. On the other hand, the recipients of the 6.5-minute booster video scored significantly higher in the three-month follow-up OOART survey. Yet, the boosted three-month follow-up survey responses were significantly lower compared to the post-OOART survey scores, averaging between the pre- and post-OOART scores. Taken together, the booster video approach offers a novel method to improve opioid overdose response knowledge and requires further development.

## Discussion

The opioid epidemic remains a significant public health issue despite the increased awareness and availability of naloxone. Previous studies have shown that providing OOART to medical students leads to significant improvements in opioid overdose response knowledge and attitudes while reducing stigma immediately following the training [19,28,29]. As depicted by Sandhu et al. [26], however, OOART knowledge declines after three months following initial training. Confirming and building upon the previous longitudinal study, we demonstrate that anti-bias attitudes and opioid overdose response knowledge increase following the implementation of a booster aOOART video. Summarized anticipated positive attitude scores were improved compared to the non-boosted participant responses at the three-month follow-up survey. This suggests that the booster video approach adds value and should be further optimized to improve participant attitudes toward people with OUD. 

The combined attitude scores showed a slight increase among both positive (Q1-5, Q7, Q11, Q12) and negative (Q6, Q9, Q10) questions in the boosted group compared to the control group at the three-month follow-up survey time point. Positive attitude questions Q1, Q3, Q4, Q5, Q11, and Q12 (which assess participants’ willingness to help, understanding the nuances of OUD and the opioid epidemic, as well as preparedness for rapid response) specifically showed an increase, albeit insignificant, among the boosted group. These findings suggest that a booster video may be helpful in preserving positive attitudes among future clinicians. The increase in negative perceptions by participants at the three-month follow-up survey time point shown in questions Q6, Q9, and Q10 (which assess perceptions of OUD and confidence in executing rapid response) was an interesting observation. Although unexpected, these results may be explained by the confusing nature of including negative questions in the same survey and using the same rating scale as the positive questions. Furthermore, the small sample size of students responding to the three-month follow-up survey may not accurately encompass the perceptions of the wider body of students who originally received OOART.

The efficacy of the booster video is further demonstrated within the combined knowledge scores at the three-month follow-up survey time point. Here, participants who received the booster video exhibited a significant increase in their knowledge score compared to the non-boosted cohort. However, both three-month follow-up cohorts had lower survey score averages compared to scores from the post-OOART survey collected immediately after the initial OOART training. These lowered scores lead us to hypothesize that while the booster video is helping participants recall the opioid overdose response knowledge, more frequent booster videos may reinforce the OOART knowledge goals. Furthermore, questions assessing components of the opioid overdose response algorithm (K1, K3-K7) were slightly increased between the boosted and non-boosted control cohorts at the three-month follow-up time point, with K3 showing significant improvement. Knowledge questions assessing the physiology of opioid overdose reversal (K2) also slightly improved following boosting. Although these types of questions are not directly a part of the rapid response algorithm, boosting this knowledge may be useful for healthcare students and providers who may have a stronger understanding of pharmacokinetics as a result of their training.

Several studies have demonstrated the effectiveness of abbreviated booster training in knowledge retention among healthcare providers, especially in other rapid response applied skills. A 2015 randomized controlled study found that a short-term, 15-minute refresher of cardiopulmonary resuscitation (CPR) training six months following initial training may help improve chest compression retainment for up to one year [30]. They showed that the number of appropriate chest compressions administered was greater (p = 0.009) and the time spent without chest compressions was shorter (p < 0.001) in the cohort that received the refresher course. Furthermore, if OOART is expanded to provide hands-on training to students, a refresher training in conjunction with aOOART may benefit skill retention.

This study presents the first quantitative longitudinal evaluations of attitude and knowledge measures following a refresher aOOART video. This longitudinal booster training and reassessment strategy will serve to improve the DUCOM Naloxone Outreach Program’s educational outreach and improve the impact of OOART training for future clinicians. Future work should optimize the aOOART booster video delivery and coverage of knowledge and attitude questions to further improve long-term training outcomes. Furthermore, future trainings should experiment with additional recall strategies such as spaced repetition, applied skill refreshers, or other longitudinal-based learning approaches to improve knowledge retention and attitude improvement.

The study’s most significant limitation was the attrition rate between the post-OOART and three-month follow-up time point surveys. Additionally, a majority of participants who completed the pre- and post-OOART survey were first-year medical students at a single institution. Future studies should reduce longitudinal attrition and assess OOART outcomes with healthcare students, professionals, and the general public. 

## Conclusions

The ability to appropriately recognize, treat, and prevent opioid overdoses is crucial for medical students. The OOART aims to provide participants with the tools and skills needed to better care for people with OUD. The findings of this study demonstrate that a 6.5-minute OOART booster video can be used as a tool in improving long-term retention of acute opioid reversal training and naloxone pharmacokinetics. This work provides a proof of concept that an OOART booster video may be an effective method to improve long-term retention and preparedness to care for people with OUD. Moving forward, we aim to continue optimizing the delivery timing and content of the aOOART to improve attitude responses (Q1-Q12). 
